# Dietary supplementation of mulberry leaf oligosaccharides improves the growth, glucose and lipid metabolism, immunity, and virus resistance in largemouth bass (*Micropterus salmoides*)

**DOI:** 10.3389/fimmu.2025.1525992

**Published:** 2025-01-28

**Authors:** Donglai Zhou, Wenhao Zhong, Bing Fu, Erna Li, Le Hao, Qingrong Li, Qiong Yang, Yuxiao Zou, Zhenxing Liu, Fubao Wang, Sentai Liao, Dongxu Xing

**Affiliations:** ^1^ Sericultural & Agri-Food Research Institute, Guangdong Academy of Agricultural Sciences, Guangdong Key Laboratory of Agricultural Products Processing, Guangzhou, China; ^2^ Guangdong Provincial Engineering Technology Research Center of Special Aquatic Functional Feed, Foshan, China; ^3^ College of Food Science and Technology, Guangdong Ocean University, Zhanjiang, China; ^4^ Institute of Animal Health, Guangdong Academy of Agricultural Sciences, Guangzhou, China

**Keywords:** largemouth bass, growth, serum biochemistry, liver metabolism, inflammation

## Abstract

This study investigated the effects of dietary supplementation of mulberry leaf oligosaccharides (MLO) on the growth performance, serum biochemistry, glucose and lipid metabolism, antioxidant activity, liver health, and virus resistance in largemouth bass (*Micropterus salmoides*). The fish were fed with CK (basal diet), MLOL (basal diet supplemented with 0.5%MLO), and MLOH (basal diet supplemented with 1.0% MLO) for 80 days, and then subjected to a 21-day viral challenge experiment. The results showed that MLO supplementation had no adverse effect on the weight gain rate, specific growth rate, feed intake, and condition factor (*P >* 0.05), but significantly decreased the feed conversion rate and viscerosomatic index (*P<* 0.05). Moreover, the MLOL and MLOH group had significantly lower contents of triglyceride, blood glucose, and malondialdehyde and activities of serum alanine aminotransferase and aspartate aminotransferase, while significantly higher levels of serum and liver total superoxide dismutase and lower levels of glutathione than the CK group (*P*< 0.05). MLO supplementation significantly up-regulated the relative expression of glycolytic genes *gk* and *pfk* and lipid catabolism genes *ppar-α* and *cpt-1*, while obviously down-regulated that of *acc*, *fas*, and *dgat* related to fatty acid synthesis in the liver tissue (*P<* 0.05). In terms of liver health, MLO supplementation significantly up-regulated the relative expression of anti-inflammatory cytokines *il-10* and *tgf-β*, while decreased that of pro-inflammatory cytokines *nf-κb*, *il-8*, and *tnf-α* in the liver tissue (*P<* 0.05). The viral challenge test showed that MLO supplementation significantly improved the survival rate of *M. salmoides* after largemouth bass ranavirus (LMBV) infection. Dietary MLO supplementation promoted liver glucose and lipid metabolism, and improved the immunity and resistance of *M. salmoides* to LMBV by regulating the PPAR signaling way and inhibiting the NF-kB signaling pathway. The appropriate addition amount of MLO to the diet was determined to be 1.0%.

## 1 Introduction

Largemouth bass (*Micropterus salmoides*) is an important economic fish species in the world as well as one of the most important freshwater economic fish in China. According to statistics, the production of *M. salmoides* in China exceeded 700,000 tons in 2021 (1). Breeding of *M. salmoides* provides a large amount of high-quality animal protein and brings great economic benefits in China. However, with the expansion of breeding scale and continuous increase in breeding density, the breeding environment has been gradually deteriorated, and the problem of diseases has become increasingly prominent, causing huge economic losses and seriously restricting the sustainable development of the industry (2). In order to treat and control diseases, antibiotics and chemicals are frequently used as therapeutic agents in aquaculture, which can lead to drug residues and have adverse effects on the safety of the environment, humans, and animals (3–5). At present, China has banned the addition of antibiotics in feed, and “reducing resistance to replace resistance” has become a new trend. Therefore, development of environment-friendly and safe alternatives to antibiotics has become a hot research topic. Among them, functional additives with preventive effects such as prebiotics and plant extracts have attracted great research attention (6).

Prebiotics are organic substances that are not directly digested and absorbed by the host, but can selectively promote the growth or activity of a few beneficial bacteria in the colon, thereby improving the health of the host (7, 8). With continuous progress in research on the functions and mechanisms of prebiotics, the application of oligosaccharides in aquaculture animals is becoming increasingly popular (9, 10). Previous research has demonstrated that dietary oligosaccharides can trap pathogenic bacteria and prevent their access to gut mucosa, and improve the activities of digestive enzymes and modulation of gut microbiota, so as to improve the growth and gut health of the reared fish (11, 12). Besides, oligosaccharides can act as immunostimulants in various fish species and significantly change the disease resistance (13, 14).

Mulberry leaf, a component of traditional Chinese medicine rich in polysaccharides, is widely grown in China (15, 16). Mulberry leaf oligosaccharides (MLO) can be obtained through enzymatic hydrolysis of mulberry leaf polysaccharides (MLP) (17). *In vitro* experiments have shown that MLO can better promote the proliferation of *Bifidobacterium* and *Lactobacillus* than glucose or galacto-oligosaccharides. In addition, bacterial cultures inoculated with MLO have higher acetic acid and lactic acid concentrations (18). A recent study has revealed that dietary supplementation of MLO can effectively reduce blood glucose level in type 2 diabetic mice (19). However, there has been limited relevant information on aquatic species. For example, *Ramulus mori* oligosaccharides could increase the abundance of Fusobacterium and *Cetobacterium* in the intestine and improve liver morphology, thereby improving the antiviral ability of *M. salmoides* (20). However, it remains unclear whether MLO can improve the immune and antioxidant capacity and glucose/lipid utilization and metabolism of *M. salmoides*. Therefore, this study aims to explore the effects of MLO supplementation on the growth performance, antioxidant capacity, glucose and lipid metabolism, liver health, and antiviral ability of *M. salmoides*, which may help improve the health outcomes in aquaculture.

## 2 Materials and methods

### 2.1 Preparation of MLO

MLP was isolated and extracted using the method as described previously (17). Briefly, defatted mulberry leaf powder was extracted with water at 80°C for 4 h at a ratio of 1: 30 (w/v), evaporated and concentrated at 50°C using a rotary evaporator (EYELA N-1100, Tokyo Rikakikai Co. Ltd, Tokyo, Japan), precipitated with 4 volumes of absolute ethanol for 12 h, and then centrifuged at 10,000× g for 10 min. The precipitate was collected and dissolved. The polysaccharide solution (50 mg/mL) was then mixed with Sevage reagent (1-butanol/chloroform, v/v = 1:4) at a ratio of 4: 1 (v/v). The mixture was shaken thoroughly for 30 min and then centrifuged at 10,000× g for 5 min. The aqueous phase separated from the supernatant was added to a quarter of its volume of the Sevage reagent. This process was repeated until the solution presented no absorption peak at 250–280 nm on a UV spectrophotometer (UV-1800, Shimadzu, Kyoto, Japan). The deproteinized liquid was further freeze-dried to obtain MLP and the phenol-sulfuric acid method was used to determine the purity. For production of the enzymatic hydrolysate containing MLO, the MLP was incubated with 1500 U/mL *β*-dextranase (Shanghai Ryon Biologcial Technology Co., Ltd., Shanghai, China) at 51°C for 4 h. Then, the hydrolysate was placed in a boiling water bath for 10 min to terminate the reaction and then centrifuged at 4000 r/min for 10 min to remove *β*-dextranase (the enzymatic optimization is not shown here). The supernatant was collected and freeze-dried to obtain MLO.

### 2.2 Molecular weight determination

The molecular weight (Mw) of MLO was determined by gel permeation chromatography (GPC) in a highperformance liquid chromatography system (LC-2050, Shimadzu, Japan) coupled with RID-20A differential refractive index detector, using a PL aquagel-OH MIXED-M column (7.5 × 300 mm, 8 μm, Agilent, USA). The MLO powder was prepared as a 10 mg/mL aqueous solution and filtered through a 0.22 μm Millipore filter. An aliquot of 20 μL sample solution was injected and eluted at a flow rate of 1.0 mL/min and a column temperature of 35°C with 0.2 mol/L NaNO_3_ as the mobile phase. PEG of 1892 Da, 5121 Da, 10057 Da, 20552 Da, and 41531 Da were used as reference substances to calculate the Mw of MLO.

### 2.3 Diet preparation

All ingredients and proximate composition of the experimental diets are shown in **Table 1**. Fish meal, soybean meal, and peanut bran were the main protein sources; fish oil, soybean oil, and soybean phospholipid were the main fat sources; and wheat flour was the main carbohydrate source. Then, 0% (CK), 0.5% MLO (MLOL), and 1% MLO (MLOH) were supplemented to the basal feed to prepare three isonitrogenous and isolipidic diets. All ingredients were ground through a 250 μm mesh, mixed thoroughly, modulated by 102°C water vapor for 8 min, and then expanded at 95°C by a twin-screw extruder (TDSP120*2-120KW, China), cut into 3 mm (diameter), dried at 75°C and stored at –20°C until use.

**Table 1:** Ingredients and proximate composition (g/kg DM) of the experimental diets.

Item	Diets^1^		
	CK	MLOL	MLOH
Ingredients			
Fish meal	350.0	350.0	350.0
Soybean meal	151.0	151.0	151.0
Peanut bran	126.0	126.0	126.0
Corn gluten meal	80.0	80.0	80.0
Spray-dried blood cells	20.0	20.0	20.0
Wheat flour	130.0	130.0	130.0
Squid paste	20.0	20.0	20.0
Yeast extract	20.0	20.0	20.0
Monocalcium phosphate	15.0	15.0	15.0
Cellulose	20.0	15.0	10.0
MLO	0.0	5.0	10.0
Fish oil	20.0	20.0	20.0
Soybean oil	20.0	20.0	20.0
Soybean phospholipid	20.0	20.0	20.0
Vitamin premix^2^	1.0	1.0	1.0
Mineral Premix^3^	3.0	3.0	3.0
Choline chloride	4.0	4.0	4.0
Nutrient levels^4^ (air-dry basis)			
Dry matter	908.4	901.8	898.6
Crude protein	485.5	485.0	486.0
Crude lipid	105.0	104.6	105.5
Ash	168.0	169.0	166.0
Crude fiber	7.5	7.3	7.2
Gross energy(MJ kg^-1^)	19.8	19.8	19.9
Nitrogen-free extract	234.0	234.1	235.3
Lysine	33.5	33.8	33.4
Methionine	11.5	11.3	11.5

^1^CK, basal diet; MLOL, supplemented with 0.5%MLO; MLOH, supplemented with 1.0%MLO.

^2^Each kilogram of vitamin premix contains vitamin A 66,666,666.7 IU, vitamin D 400,000,000 IU, vitamin E 1 g, vitamin K 2 g, vitamin B_1_ 5_ g_, vitamin B_2_ 5_ g_, vitamin B_6_ 5_ g_, vitamin B_12_ 1_ g_, calcium pantothenate 20 g, folic acid 10 g, biotin 1 g, niacin 20 g, choline chloride 200 g, defatted rice bran 700 g.

^3^Each kilogram of mineral premix contains CuCO_3_ 4 g, FeC_6_H_5_O_7_ 15 g, MgO 26 g, MnSO_4_ 5 g, KCl 250 g, ZnSO_4_ 50 g, NaCl 50 g, Zeolite powder 600 g.

^4^Nutrient levels were measured values.

### 2.4 Experimental design and feeding trial

Prior to the feeding trial, fish were fed with the basal diet for two weeks to allow acclimation to diet and conditions in an indoor circulating aquaculture system of the Research Institute of Sericulture and Agricultural Product Processing, Guangdong Academy of Agricultural Sciences (Guangzhou, China). After 24 h of fasting, a total of 450 *M. salmoides* (initial body weight 26.89 ± 1.16 g) were randomly distributed into nine tanks (350 L) with 50 fish in each tank and three tanks in each group. The experiment involved a completely randomized design. Fish were fed twice a day (09: 00 and 16: 30) to achieve an apparent state of satiety, over the 80 days of experiment. The water temperature was kept at 23.0–29.0°C, pH = 7.0–8.2, dissolved oxygen > 6.0 mg/L, ammonia ≤ 0.02 mg/L and nitrite ≤ 0.1 mg/L. Mortality was observed daily and the dead fish were weighed and recorded. The animal husbandry and handling protocols used in this study have been approved by the Animal Care and Use Committee, Guangdong Academy of Agricultural Sciences (GDAAS 258/2019).

### 2.5 Sampling

At the end of feeding trial, the fish were starved for 24 h before weighing. All fish were anesthetized with 40 mg/L tricaine methanesulfonate (MS-222, Sigma, USA) before sampling. Fish in each tank were counted and weighted to measure the final body weight, weight gain rate, specific growth rate, feed intake, feed conversion ratio, and condition factor. The blood of nine fish per tank was drawn from the caudal vein, kept at 25°C, centrifuged at 3,000 rpm for 15 min, and the supernatant was stored at –80°C for the analysis of serum biochemistry and antioxidant index. Afterwards, the fish were slaughtered and dissected using a sterile scalpel by cutting the belly on ice for determination of the viscerosomatic index and hepatosomatic index.

The livers of three fish per tank were collected and stored at –20°C for the analysis of antioxidant and immunity parameters. The livers of another three fish per tank were collected and immediately stored at –80°C for glucose and lipid metabolism, RNA extraction, and qPCR analysis.

### 2.6 Laboratory analyses

The nutrient composition and amino acids of the diets were analyzed using AOAC method (21) and determined by high-performance liquid chromatography (22), respectively.

The contents of blood glucose (GLU), low-density lipoprotein cholesterol (LDL-C), high-density lipoprotein cholesterol (HDL-C), total cholesterol (TC), triglycerides (TG), aspartate aminotransferase (AST), alanine aminotransferase (ALT), reduced glutathione (GSH), malondialdehyde (MDA), total antioxidant capacity (T-AOC), and total superoxide dismutase (T-SOD) in the serum or liver were determined with commercial kits (Nanjing Jiancheng Bioengineering Institute Nanjing, China).

The livers (0.5 cm × 0.5 cm × 0.5 cm) of three fish per tank were sampled, rinsed by 0.65% saline (4°C), fixed immediately in the 10% buffered formalin solution for 24 h. The fixed liver tissue was embedded in paraffin, and paraffin sections were made and stained with hematoxylin eosin (HE) using the standard histological techniques. The slices were scanned using a panoramic slicing digital scanner (PANNORAMIC-1000, 3DHISTECH), and the CaseViewer 2.2 (3DHISTECH) software was used to capture tissue photos of the slices (23).

Total RNA was extracted from each liver sample using Trizol reagent (TIANGEN, China) and the RNA quality was tested using Genios plus (Synergy LX, BioTek, USA). An aliquot of 1 mg total RNA was used for reverse transcription. The cDNA was synthesized by RevertAid Reverse Transcriptase (TIANGEN, China) using random primers. The RT-qPCR was performed according to standard protocols in a quantitative thermocycler (MiniOption TM, Bio-Rad, USA). The amplification volume was 10 μL, containing 0.3 μL of the respective primers, 1 μL of cDNA product, 5 μL of SYBR green color qPCR Master Mix (TIANGEN, China), and 3.4 μLof ddH_2_O. The specific primers used for *il-8*, *il-10*, *nf-kb*, *tnf-α*, *tgf-β*, *gk*, *pfk*, *acc*, *cpt1*, *fas*, *dgat*, and *ppar-α* genes have been reported previously (24–27). The details of the primers and the PCR amplification conditions used for RT-qPCR are shown in **Table 2**. After the reaction, melting curve analysis was conducted to confirm the presence of a single PCR product in the reaction. The gene expression levels were calculated by the 2^-ΔΔCt^ method (28) with *β-actin* as the reference gene, and the experiment was performed in triplicate.

**Table 2:** Primer sequences for RT-qPCR in the experiment.

Gene	Forward primer (5′−3′)	Reverse primer (5′−3′)	Length (bp)	Sources	Amplification efficiency (%)
*il-8*	ACTTCTCCTGGCTGCTCTG	ACTTCTCATTTGGTTTGACACA	170	XM_038704089.1	96.4
*il-10*	CGGCACAGAAATCCCAGAGC	CAGCAGGCTCACAAAATAAACATCT	113	XM_038696252.1	102.5
*nf-kb*	AGAAGACGACTCGGGGATGA	GCTTCTGCAGGTTCTGGTCT	119	XM_038699793.1	96.4
*tnf-α*	AAATAGTGATTCCTCAAGACGG	TGAACAGTATGGCTCAGATGG	126	XM_038723994.1	106.3
*tgf-β*	GCTTCAGTTTCGGCATTT	TCTCCGTGGAGCGTTTT	186	XM_038693206.1	94.5
*gk*	CTCGCTCTGCTCGTATGT	CTCCCTTCCTCCGACTG	208	XM_038703172.1	101.3
*pfk*	TGGGCTATGATACAAGAGTGA	CCATTAGAGGCAGACGAAC	189	XM_038720351.1	98.6
*acc*	AAGTCCAAGAGGGCACG	ACTGGGAGTCCGCAAAT	173	XM_038704615.1	102.4
*cpt-1*	GATGTTTTATGACGGGCGG	TAGGTTTCACGAGCATTGGC	156	XM_038705335.1	103.1
*ppar-α*	CCACCGCAATGGTCGATATG	TGCTGTTGATGGACTGGGAAA	144	XM_038705497.1	103.6
*β-actin*	TTCACCACCACAGCCGAAAG	TCTGGGCAACGGAACCTCT	179	KJ669298.1	102.2
*fas*	GCCCTTGACTCATTCCG	GCCCTTGACTCATTCCG	234	XM_038735140.1	102.1
*dgat*	GCAACATCAAGCCGTCCGACTC	AGCACAGCGAGCCAGAGGTAAT	176	XM_038705876.1	105.3

*il-8*, interleukin-8; *il-10*, interleukin-10; *nf-kb*, nuclear factor-kB; *tnf-α*, tumor necrosis factor*-α*; *tgf-β*, transforming growth factor-*β*; *gk*, glucokinase; *pfk*, phosphofructokinase; *acc*, acetyl-CoA carboxylase; *cpt-1*, carnitine palmitoyl transferase 1; *ppar-α*, peroxisome proliferators-activated receptors *α*; *fas, fatty acid synthetase; dgat*, diacylglycerol acyltransferase.

### 2.7 Challenge test

The largemouth bass ranavirus (LMBV) was isolated from diseased largemouth bass and stored in our laboratory (Guangdong Provincial Key Laboratory of Livestock Disease Prevention, Institute of Animal Health, Guangdong Academy of Agricultural Sciences). After 80 days of feeding, 20 fish were randomly selected from each group for challenge experiment. Each fish was injected with 100 μL 4 × 10^5^ TCID_50_/mL of LMBV infected cell suspernatant through the intraperitoneal cavity. The virus dose was based on the results of our previous experiments (20). The mortality rate was recorded daily for 21 days. The relative percent survival (RPS) was calculated according to the following method.

### 2.8 Data calculation and statistical analysis


$$\begin{array}{c} \text{Weight gain rate }\left(\text{WGR, }\%\right)=(\text{final body weight }\left(\text{FBW, g}\right)\\ –\text{initial body weight }\left(\text{IBW, g}\right))/\text{IBW}\left(\text{g}\right)\times 100 \end{array}$$

$$\begin{array}{c} \text{Specific gain rate }\left(\text{SGR, }\%/\text{d}\right)\\ =\left(\text{ln FBW }\left(\text{g}\right)–\text{ln IBW }\left(\text{g}\right)\right)/\text{days}\times 100 \end{array}$$

$$\begin{array}{c} \text{Feed intake }\left(\text{FI, g/fish}\right)=\text{feed consumed }\left(\text{g}\right)/\\ \left(\text{final fish number}+\text{initial fish number}\right)/2 \end{array}$$

$$\begin{array}{c} \text{Feed conversion rate }\left(\text{FCR}\right)\\ =\text{feed intake }\left(\text{g}\right)/\left(\text{FBW }\left(\text{g}\right)–\text{IBW }\left(\text{g}\right)\right) \end{array}$$

$$\begin{array}{c} \text{Condition factor }\left(\text{CF, }\%\right)\\ =\text{FBW }\left(\text{g}\right)/\text{final body length }\left(\text{cm}\right))\times 100 \end{array}$$

$$\begin{array}{c} \text{Hepatosomatic index }\left(\text{HSI, }\%\right)\\ =\text{Hepatosoma weight }\left(\text{g}\right)/\text{body weight }\left(\text{g}\right)\times 100 \end{array}$$

$$\begin{array}{c} \text{Viscerosomatic index }\left(\text{HVI, }\%\right)\\ =\text{visceral weight }\left(\text{g}\right)/\text{body weight }\left(\text{g}\right)\times 100 \end{array}$$

$$\begin{array}{c} \text{Relative percent survival }\left(\text{RPS, }\%\right)\\ =(\text{percent mortality in control group}\\ –\text{percent mortality in the treatment group})\\ /\text{percent mortality in control group}\times 100 \end{array}$$

The data were analyzed using one-way ANOVA with Duncan’s method for multiple comparisons between groups. Orthogonal polynomial contrast was used to estimate the linear and quadratic effects of various amounts of MLO added. All data were analyzed using SPSS. The significance level of differences was set at *P<* 0.05 and the results were expressed as Mean ± SE. GraphPad Prism8.0 software was used to analyze the survival curves, and Log-rank (Mantel-Cox) was used for statistical difference analysis.

## 3 Results

### 3.1 Molecular weight of MLO

**Figure 1:**
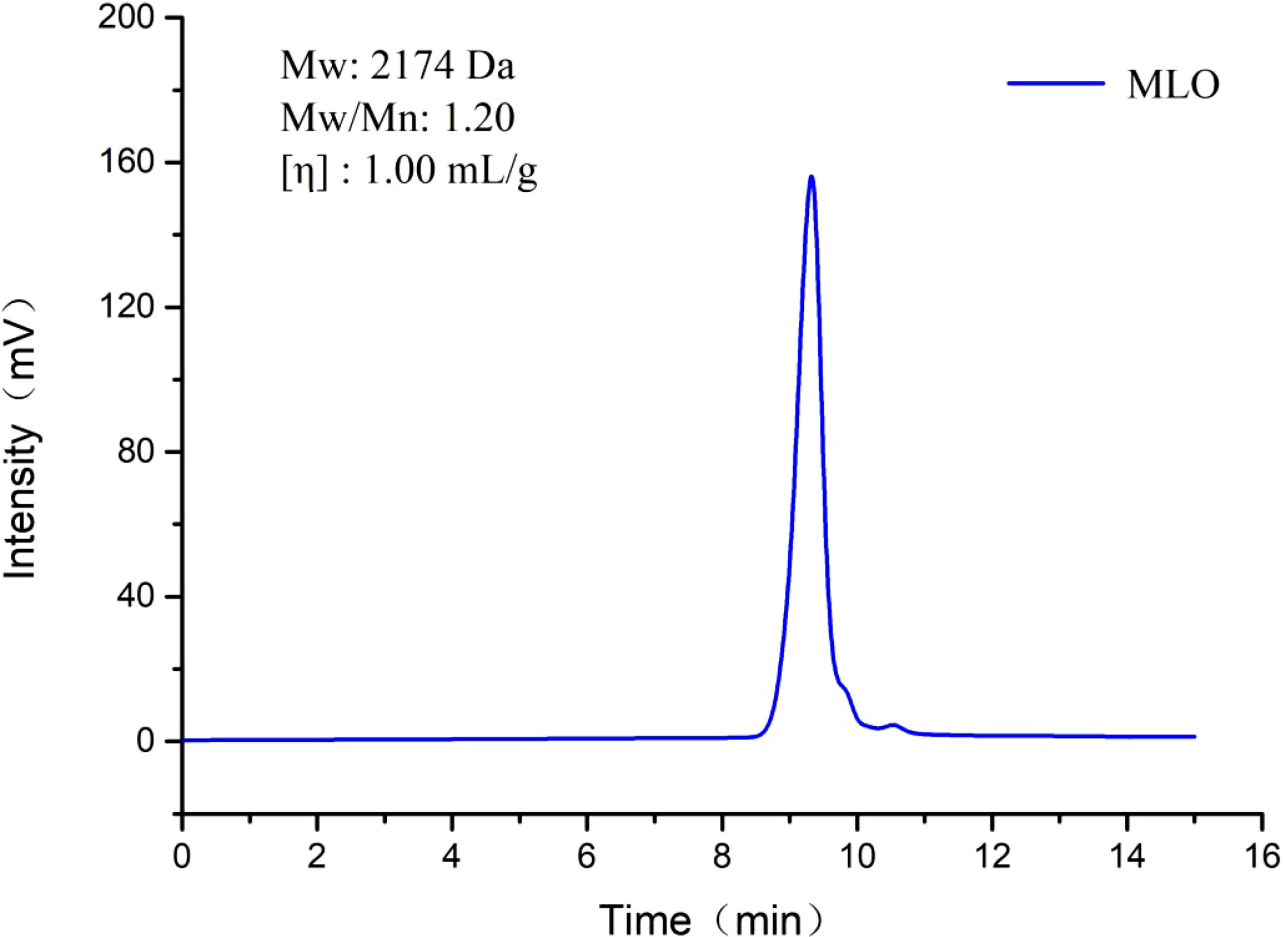
Molecular weight distribution diagram of MLO. Mw, average molecular weight; Mn, number-average molecular weight; Mw/Mn, polydispersity index; [η], intrinsic viscosity.

GPC analysis was conducted to determine the average molecular weight (Mw), number-average molecular weight (Mn), and polydispersity index (Mw/Mn) for the MLO. As shown in **Figure 1**, the GPC profile of MLO displayed a single symmetrical and sharp peak. The values of Mw, Mw/Mn, and intrinsic viscosity [η] were determined to be 2174 Da, 1.20, and 1.00 mL/g, respectively.

### 3.2 MLO supplementation has no adverse effect on the growth performance of largemouth bass

After the feeding experiments, no pathology or abnormal phenomenon was observed in all groups. The effects of dietary MLO level on FBW, WGR, SGR, FI, FCR, CF, HVI, and HSI are shown in **Table 3**. There was no significant difference in FBW, WGR, SGR, FI, and CF among all groups (*P* > 0.05). However, compared with the CK group, the MLOL and MLOH group showed significant decreases in FCR and HVI (*P<* 0.05). Moreover, the MLOH group had significantly lower HSI than the CK group (*P<* 0.05). With increasing level of MLO added to the diet, there were significant decreases in FCR (linear or quadratic, *P*< 0.05) and VSI and HSI (linear, *P*< 0.05).

**Table 3:** Growth performance of *M. salmoides* fed with the experimental diets.

Items^2^	Diets^1^			SEM^2^	*P*-value		
	CK	MLOL	MLOH		ANOVA	Linear	Quadratic
Initial body weight, g	28.53	26.00	26.13	1.97	0.407	0.269	0.464
Final body weight, g	173.54	166.98	163.15	5.50	0.241	0.108	0.785
Weight gain rate, %	515.78	543.12	524.61	25.77	0.585	0.743	0.344
Specific growth rate, %/d	3.98	4.12	4.06	0.11	0.491	0.498	0.338
Feed intake, g/fish	203.02	192.99	189.28	6.59	0.179	0.082	0.600
Feed conversion rate	0.89^a^	0.71^c^	0.78^b^	0.01	<0.001	<0.001	<0.001
Condition factor, g/cm^3^	2.28	2.27	2.20	0.06	0.504	0.296	0.648
Viscerosomatic index, %	9.14^a^	8.68^b^	8.41^b^	0.12	0.002	0.001	0.381
Hepatosomatic index, %	2.24^a^	2.14^a^	1.87^b^	0.05	0.001	<0.001	0.075

^1^CK, basal diet; MLOL, supplemented with 0.5% MLO; MLOH, supplemented with 1.0% MLO.

^a,b,c^Different letters with a row indicate significant differences (*P*< 0.05).

^2^SEM, Standard error of the mean.

### 3.3 MLO improves the serum biochemical parameters of largemouth bass

The biochemical parameters in the serum of *M. salmoides* after feeding with MLO diets for 80 days are presented in **Table 4**. The MLOL and MLOH group had significantly lower TG and GLU contents as well AST and ALT activities than the CK group (*P<* 0.05), but showed no significant change in the contents of TC, LDL-C, and HDL-C in the serum (*P* > 0.05). With increasing level of MLO added to the diet, there were significant decreases in the serum TG content and ALT activity (linear or quadratic, *P*< 0.05), GLU content (quadratic, *P*< 0.05), and ALT activity (linear, *P*< 0.05).

**Table 4:** Serum biochemical parameters of *M. salmoides*.

Items^3^	Diets^1^			SEM^2^	*P*-value		
	CK	MLOL	MLOH		ANOVA	Linear	Quadratic
TG (mmol/L)	23.68^a^	17.03^b^	19.05^b^	1.16	0.003	0.007	0.005
TC (mmol/L)	14.19	11.07	12.23	1.28	0.123	0.176	0.102
GLU (mmol/L)	3.37^a^	2.59^b^	2.82^b^	0.23	0.005	0.093	0.002
LDL-C (mmol/L)	6.39	5.87	6.41	0.39	0.362	0.951	0.171
HDL-C (mmol/L)	9.43	5.76	8.87	1.85	0.184	0.774	0.079
ALT (U/mL)	45.36^a^	41.27^b^	40.19^b^	0.80	<0.001	<0.001	0.004
AST (U/mL)	32.33^a^	26.10^b^	26.11^b^	1.00	0.005	0.002	0.135

^1^CK, basal diet; MLOL, supplemented with 0.5%MLO; MLOH, supplemented with 1.0%MLO.

^a,b^Different letters with a row indicate significant differences (*P<* 0.05).

^2^SEM, Standard error of the mean.

^3^TG, triglycerides; TC, total cholesterol; GLU, blood glucose; LDL-C, low-density lipoprotein cholesterol; HDL-C, high-density lipoprotein cholesterol; ALT, alanine aminotransferase; AST, aspartate aminotransferase.

### 3.4 MLO modulates glucose and lipid metabolism in the liver

**Figure 2:**
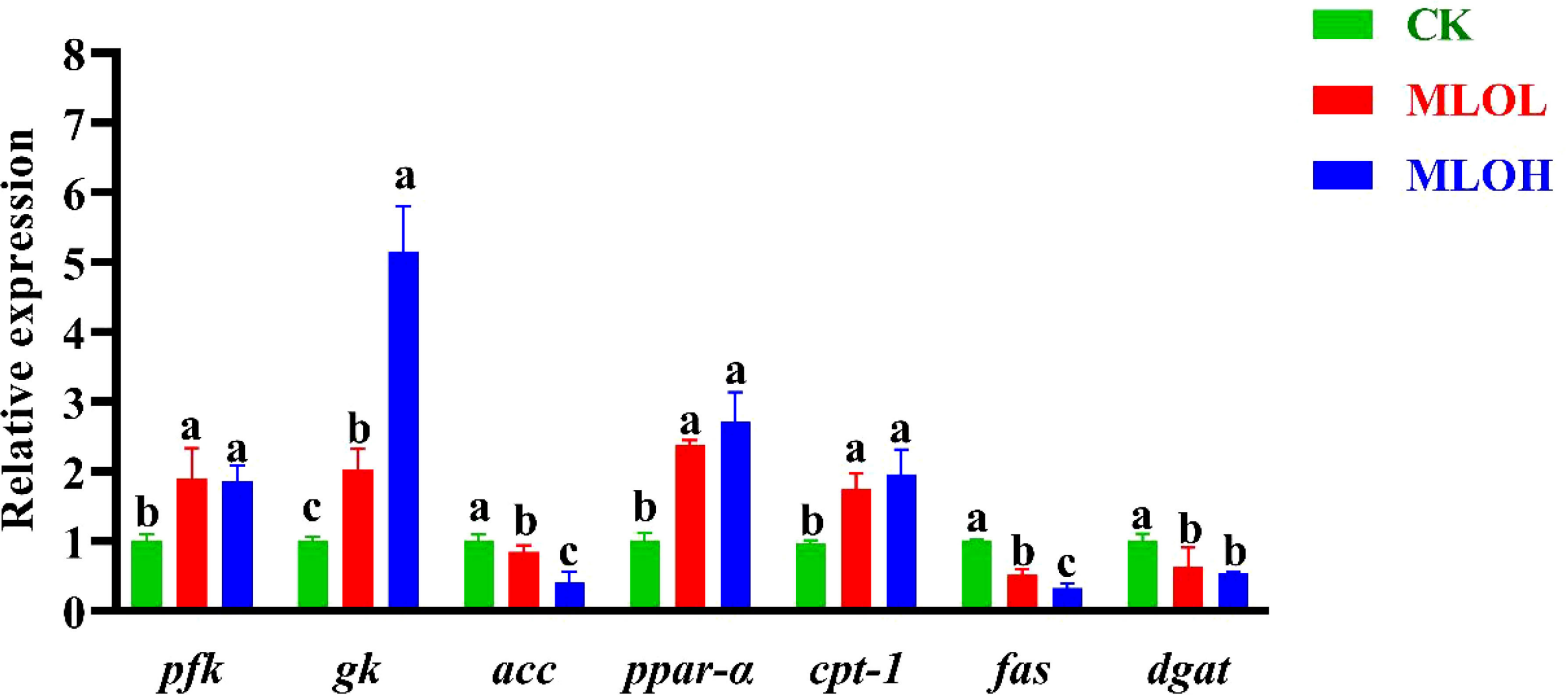
mRNA expression of *pfk*, *gk*, *acc*, *ppar-α, cpt-1, fas* and *dgat* in the liver of *M. salmoides*. CK, basal diet; MLOL, supplemented with 0.5% MLO; MLOH, supplemented with 1.0% MLO. Different letters above the bars denote significant differences among diets (*P*< 0.05).

The liver plays an important role in regulating blood glucose homeostasis (29). The MLOL and MLOH groups showed significant increases in the expression of genes related to glycolysis (*pfk* and *gk*) and lipid catabolism (*ppar-α* and *cpt1*), while significant decreases in that of genes associated with fatty acid synthesis (*acc*, *fas*, and *dgat*) relative to the CK group (*P*< 0.05) (**Figure 2**).

### 3.5 Feeding of MLO enhances the antioxidant capacity of largemouth bass

In the serum, the MLOL and MLOH group had significantly higher activities of T-SOD and GSH (*P<* 0.05), while no significant difference in the activity of T-AOC (*P* > 0.05) compared with the CK group. MLO feeding significantly increased the activities of T-SOD, GSH, and T-AOC in the liver, but obviously reduced the MDA content in the serum and liver compared with the CK (*P<* 0.05) (**Table 5**). With increasing level of MLO added to the diet, there were significant increases in serum and liver T-SOD and GSH activities (linear or quadratic, *P*< 0.05), a significant decrease in serum MDA content (linear, *P*< 0.05), a significant reduction of liver MDA content (linear or quadratic, *P*< 0.05), and a significant increase in liver T-AOC capacity (linear, *P*< 0.05).

**Table 5:** Serum and liver antioxidant parameters of *M. salmoides*.

Items^3^	Diets^1^			SEM^2^	*P*-value		
	CK	MLOL	MLOH		ANOVA	Linear	Quadratic
Serum							
T-SOD (U/mL)	49.39^c^	53.85^b^	64.08^a^	0.84	<0.001	<0.001	0.008
GSH (U/mL)	11.93^b^	19.88^a^	18.18^a^	1.91	0.014	0.017	0.027
T-AOC (U/mL)	0.45	0.49	0.52	0.03	0.157	0.064	0.909
MDA (nmol/mL)	22.43^a^	16.61 ^b^	12.41^b^	1.75	0.004	0.001	0.609
Liver							
T-SOD (U/mgprot)	11.60^c^	11.93^b^	14.02^a^	0.10	<0.001	<0.001	<0.001
GSH (U/mgprot)	9.59^b^	10.86^a^	11.04^a^	0.20	0.001	<0.001	0.019
T-AOC (U/mgprot)	0.04 ^c^	0.05 ^b^	0.06^a^	0.002	<0.001	<0.001	0.642
MDA (nmol/mgprot)	0.85^a^	0.50^b^	0.54^b^	0.02	<0.001	<0.001	<0.001

^1^CK, basal diet; MLOL, supplemented with 0.5%MLO; MLOH, supplemented with 1.0%MLO.

^a,b,c^Different letters with a row indicate significant differences (*P<*0.05).

^2^SEM, Standard error of the mean.

^3^T-SOD, total superoxide dismutase; GSH, reduced glutathione; T-AOC, total antioxidant capacity; MDA, malondialdehyde.

### 3.6 MLO supplementation inhibits inflammation in the liver

**Figure 3:**
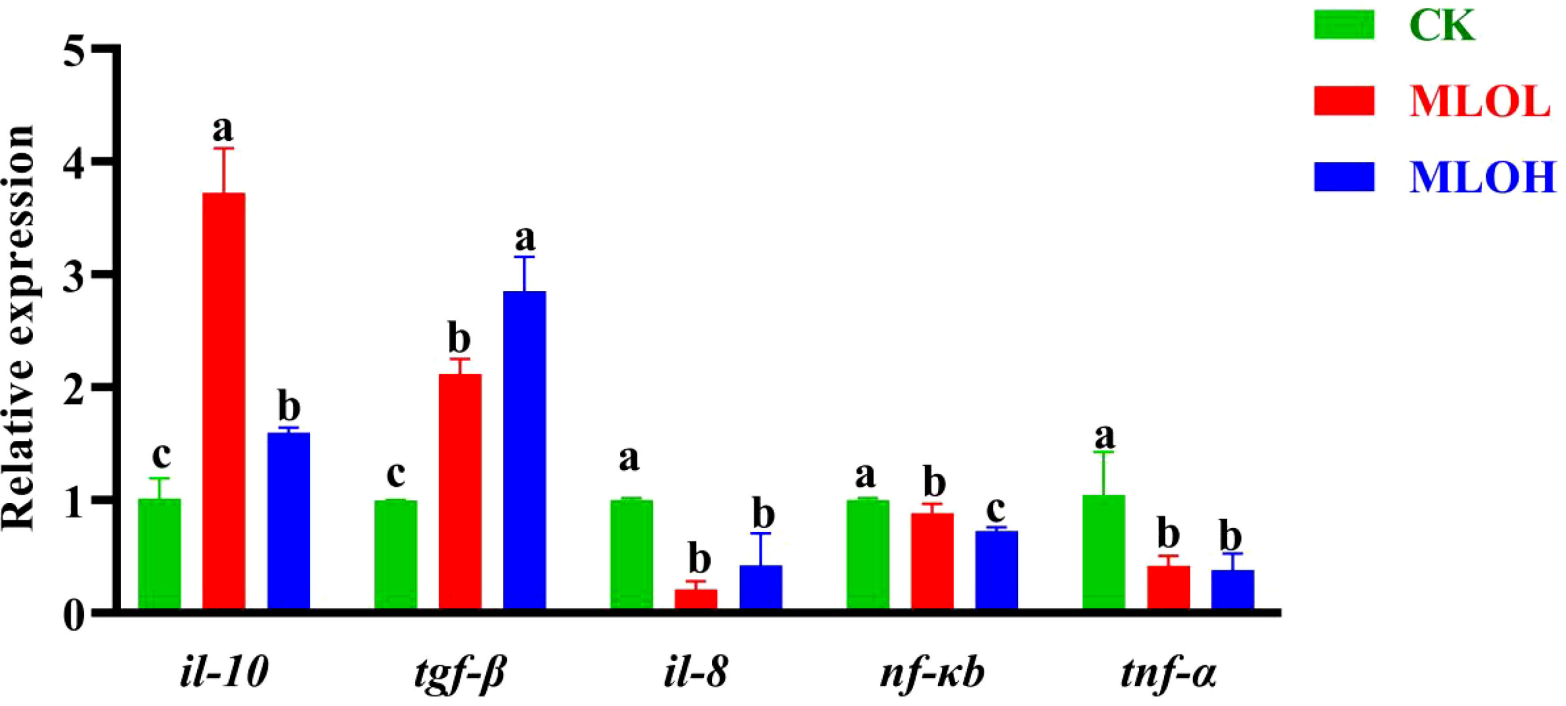
mRNA expression of *il-10*, *tgf-β*, *il-8*, *nf-kb*, and *tnf-α* in the liver of *M. salmoides*. CK, basal diet; MLOL, supplemented with 0.5%MLO; MLOH, supplemented with 1.0% MLO. Different letters above the bars denote significant differences among diets (*P*< 0.05).

The expression levels of immune-related genes are shown in **Figure 3**. Compared with the CK, MLO supplementation significantly up-regulated the relative expression of anti-inflammatory cytokines *il-10* and *tgf-β*, while down-regulated that of pro-inflammatory cytokines *nf-κb*, *il-8*, and *tnf-α* in the liver (*P*< 0.05) (**Figure 3**).

### 3.7 MLO supplementation maintains the structural integrity of the liver

**Figure 4:**
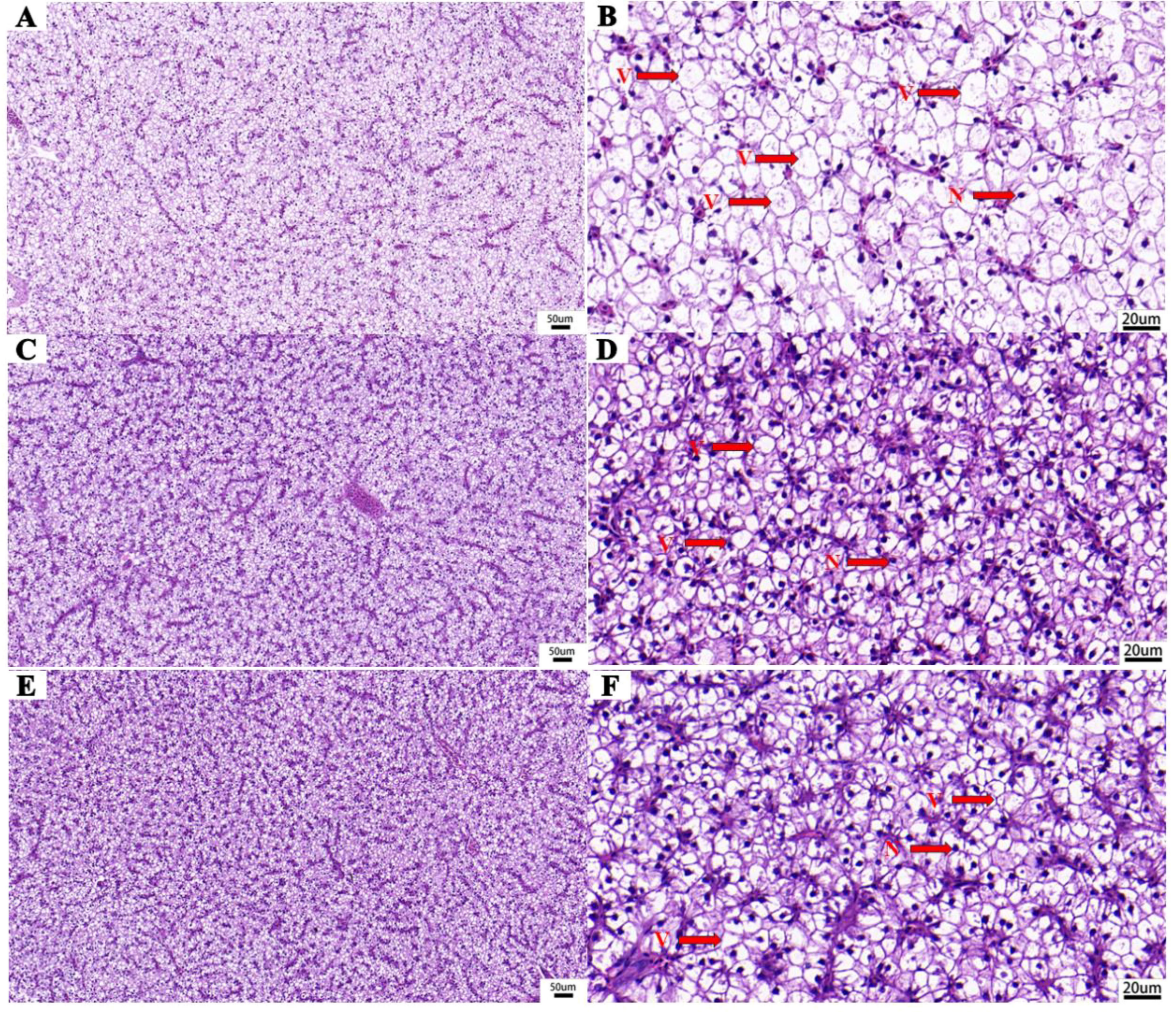
Liver histomorphology (H&E staining) of *M. salmoides*. **(A, B)** basal diet; **(C, D)** supplemented with 0.5% MLO; **(E, F)** supplemented with 1.0% MLO. V, cytoplasmic vacuolation; N, nucleus.

The liver histomorphology is shown in **Figure 4**. The liver cells of the CK group showed obvious vacuolation and nuclear displacement, while those of MLOL and MLOH group exhibited much less obvious cell vacuolization and nuclear displacement.

### 3.8 MLO supplementation improves the resistance of largemouth bass to LMBV infection

**Figure 5:**
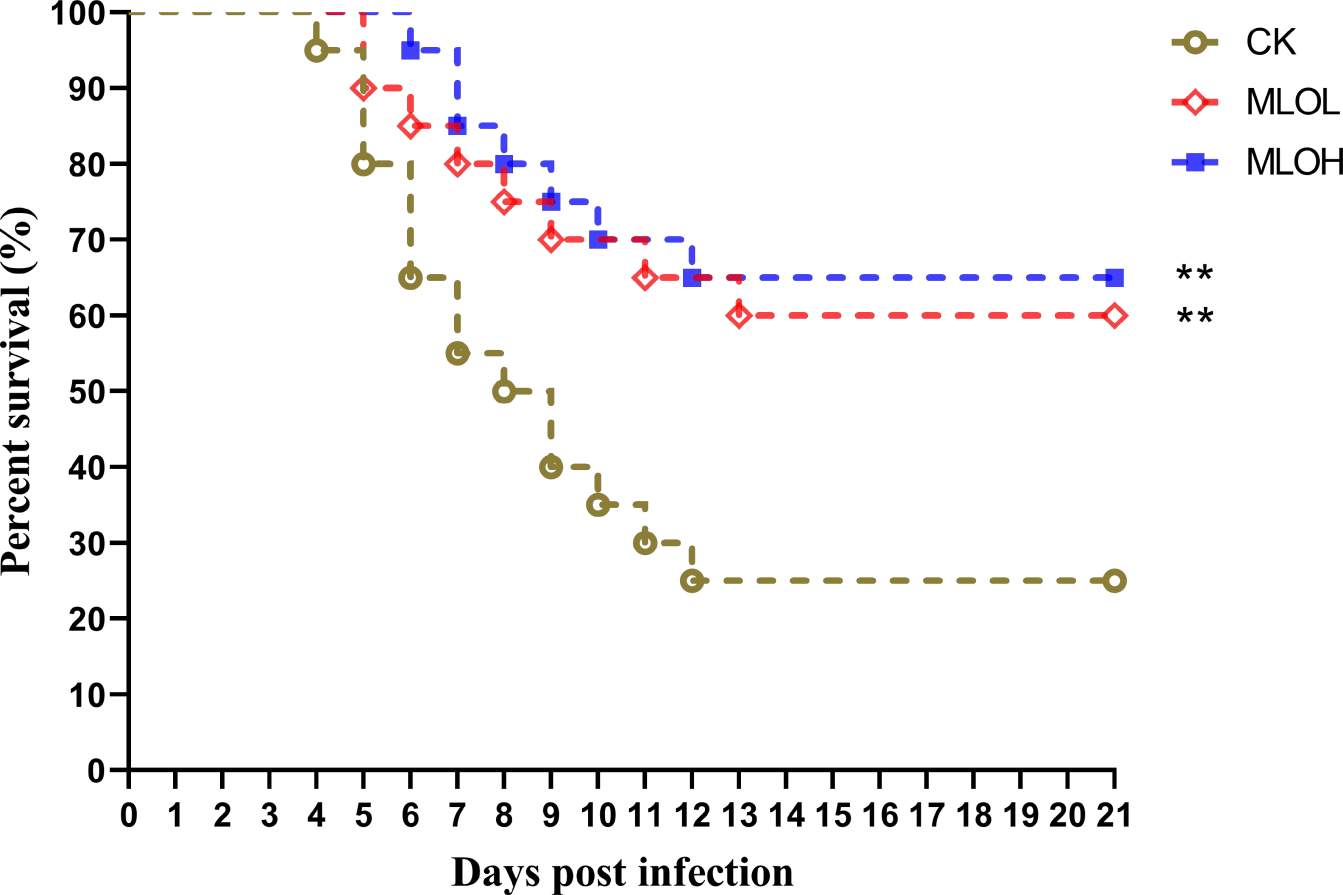
Survival rate curve analysis of *M. salmoides* after infection with LMBV. CK, basal diet; MLOL, supplemented with 0.5% MLO; MLOH, supplemented with 1.0% MLO (n = 25).

As shown in **Figure 5**, the cumulative survival rate was calculated after 21 days of LMBV infection. Compared with the CK group, the MLO groups showed significantly delayed death time of largemouth bass. There was no significant difference in the number of deaths in each group after 14 days of infection. Among different groups, the final survival rates of the CK, MLOL, and MLOH group were 25%, 60%, and 65%, respectively, indicating that MLO supplementation can significantly improve the resistance of largemouth bass to LMBV infection and enhance the survival rate (*P*< 0.05). Compared with that of the CK, the relative protection rate of MLOL and MLOH was 46.67% and 53.33%, respectively (**Figure 5**).

## 4 Discussion

### 4.1 Growth performance

To date, there have been numerous studies of the application of oligosaccharides (prebiotics) such as fructooligosaccharides, galactooligosaccharides, and isomaltooligosaccharides in aquaculture, but there has been no report about the application of MLO in aquatic animals. In the present study, diets supplemented with 0.5% and 1% MLO were found to significantly decrease the FCR and HIS, but showed no significant effect on WGR, which may be directly related to the lower feed intake of the treatment groups. Some prebiotics have also been found to have no significant effect on the growth of aquatic animals, such as short-chain fuctooligosaccharides for hybrid tilapia (*Oreochromis niloticus×O. aureus*) (14) and chito-oligosaccharide (COS) for the same species (30). Previous studies have demonstrated that oligosaccharides can significantly increase the WGR and SGR while decrease the FCR of *Oreochromis niloticus* (31), hybrid catfish (*Pangasianodon gigas* × *Pangasianodon hypophthalmus*) (32) and Caspian trout (*Salmo trutta caspius*) (33). Besides, another study has revealed that Grobiotic-A has an adverse effect on the growth of *M. salmoides* (34). These results indicate that prebiotics have different effects on the growth performance of different aquatic animals, which may be related to the type and dosage of the prebiotics used.

### 4.2 Glucose and lipid metabolism in the liver

Compared with herbivorous and omnivorous species, carnivorous fish have a poor ability to utilize starch as an energy source. Generally, digestible carbohydrate at levels of ≤15–25% is appropriate for marine and carnivorous fish, but the suitable carbohydrate level appears to be lower for *M. salmoides* (35, 36). Mulberry leaf is a Chinese medicinal component widely used to regulate blood GLU (37). Flavonoids are one of the main classes of active ingredients in mulberry leaves, which contribute to the potential to treat type II diabetes and maintain the glucose metabolism balance of *Monopterus albus* under high glucose stress (38, 39). It has been demonstrated that *M. salmoides* can strengthen the glycolysis pathway to cope with the rising of blood GLU level so as to maintain GLU metabolism homeostasis (35, 40). Another study has also revealed that mannan oligosaccharides (MOS) can decrease serum glucose and liver glycogen by increasing the activities of enzymes related to glycolysis in *O. niloticus* (41). In this study, feeding of MLO diets significantly decreased the serum GLU content in *M. salmoides*. The liver plays an important role in regulating blood GLU homeostasis (29), and this regulation involves several important pathways, such as gluconeogenesis and glycolysis, where PFK, PK, and GK are limiting enzymes in the glycolysis pathway (40). This study showed that *M. salmoides* fed with MLO diets had significantly higher mRNA expression of *pfk* and *gk*, which is similar to the result reported for *M. salmoides* (42), indicating that MLO has great potential to lower GLU.

CPT1 and ACC are key enzymes involved in lipid catabolism and fatty acid synthesis, respectively (43). PPAR-α is a transcription factor that mediates the oxidative breakdown of liver fatty acids, upregulates the expression of fatty acid transport- and oxidation-related gene *cpt-1*, and promotes the transport of long-chain fatty acids in mitochondrial *β*-oxidation, thereby improving fatty acid oxidation in mitochondria and peroxisomes (44). It has been reported that MOS can significantly reduce the TG level of *O. niloticus* and *M. salmoides* fed with high carbohydrate diets (41, 45). In this study, MLO diet up-regulated the expression of *cpt-1* and *ppar-α*, down-regulated that of fatty acid synthesis-related gene *acc*, and significantly reduced the content of TG in the serum, indicating that MLO may accelerate lipid metabolism by regulating the expression of liver metabolism-related genes, thereby reducing lipid deposition in the liver.

### 4.3 Antioxidant and immune responses

Oxidative stress and inflammatory response are interrelated processes with significant contribution to the body’s response to various stresses. There were decreases in serum GLU, AST, and ALT after dietary MLO supplementation in this study, suggesting that dietary MLO has obvious positive effects on the immune function and liver health of *M. salmoides*, since high concentrations of serum GLU, AST, and ALT are generally related to liver damage or necrosis (46, 47). It is generally believed that hepatocyte is highly sensitive to the nutritional status of fish and the quality of diet. In this study, dietary MLO significantly alleviated liver vacuolization and nuclear displacement, suggesting that it can improve the liver health of *M. salmoides*. The concentration of MDA can directly reflect the level of lipid peroxidation and the degree of endogenous oxidative damage. GSH and T-SOD are both antioxidant enzymes that assist the maintenance of a healthy cellular antioxidant state. In particular, GSH, as a substrate for glutathione peroxidase and glutathione transferase, reacts with intracellular free radicals and peroxides under the catalysis of both enzymes to maintain the normal physiological functions of cells, making it a critical enzyme for protecting cells from oxidative damage (48). T-SOD is a necessary antioxidant enzyme in all oxygen-breathing organisms, playing important roles in converting superoxide into hydrogen peroxide and removing excess active oxygen (49). Oligosaccharides have been demonstrated to have antioxidant activities in *O. niloticus* (50). Similarly, in the present study, the increase in MLO supplementation enhanced the GSH and T-SOD activities in the serum and liver while reduced the MDA content in *M. salmoides*. Therefore, MLO can enhance the antioxidant capacity of *M. salmoides.*

The inflammatory responses in animals are regulated by the NF-κB/TLRs signaling pathway (49). After activation by pro-inflammatory cytokines (such as *il-8*, *tnf-α*, and *nf-κb*), this signaling pathway can promote cellular immune response and up-regulate the expression of anti-inflammatory cytokines (such as *il-10* and *tgf-β*). This study showed that MLO supplementation remarkably up-regulated the transcription of *il-10* and *tgf-β* in the liver, whereas down-regulated that of *il-8*, *tnf-α*, and *nf-κb*. These results indicate that MLO supplementation can reduce inflammatory response by inhibiting the NF-κB signaling pathway.

### 4.4 Disease resistance

With the expansion of breeding scale, increase in breeding density, and deterioration of water environment, the problem of diseases is becoming increasingly prominent for *M. salmoides*. *M. salmoides* suffers from high mortality rates caused by viral diseases, particularly iridescent virus diseases, which poses serious threats to the aquaculture industry worldwide. *M. salmoides* infected with iridovirus generally exhibits liver and intestinal necrosis and inflammatory lesions, increased cell apoptosis, and decreased immunity (51, 52).

In the present study, MLO supplementation significantly delayed the death time of *M. salmoides* infected with LMBV compared with the CK. In addition, the cumulative mortality rate significantly decreased with increasing dietary MLO, indicating that MLO can reduce the LMBV-induced mortality rate. This may be due to the combined effect of MLO induced up-regulation of anti-inflammatory cytokines and down-regulation of pro-inflammatory cytokines, inhibition of NF-κB signaling pathway, and enhancement of the antioxidant capacity (T-SOD and GSH) in the serum and liver. Some studies have also shown that oligosaccharides can stimulate fish tissue, induce the expression of *iNOS* and produce NO, thereby killing pathogens and enhancing the innate immune response of the body (53). However, further research is still needed to clarify the mechanisms through which these oligosaccharides improve the health and immunity of *M. salmoides*.

## 5 Conclusion

In summary, MLO supplementation can significantly reduce the feed conversion rate, enhance the antioxidant capacity and liver glucose and lipid metabolism, improve the immunity by inhibiting the NF-kB signaling pathway, and increase the survival rate of *M. salmoides* infected with LMBV. This study provides a practical strategy to apply MLO in the diet for improving the immune and antioxidant abilities of *M. salmoides*.

## Data Availability

The original contributions presented in the study are included in the article, further inquiries can be directed to the corresponding authors.
